# A Differential Role for Neuropeptides in Acute and Chronic Adaptive Responses to Alcohol: Behavioural and Genetic Analysis in *Caenorhabditis elegans*

**DOI:** 10.1371/journal.pone.0010422

**Published:** 2010-05-03

**Authors:** Philippa Mitchell, Richard Mould, James Dillon, Steven Glautier, Ioannis Andrianakis, Christopher James, Amanda Pugh, Lindy Holden-Dye, Vincent O'Connor

**Affiliations:** 1 School of Biological Sciences, University of Southampton, Southampton, United Kingdom; 2 School of Psychology, University of Southampton, Southampton, United Kingdom; 3 Institute of Sound and Vibration Research, University of Southampton, Southampton, United Kingdom; Brown University, United States of America

## Abstract

Prolonged alcohol consumption in humans followed by abstinence precipitates a withdrawal syndrome consisting of anxiety, agitation and in severe cases, seizures. Withdrawal is relieved by a low dose of alcohol, a negative reinforcement that contributes to alcohol dependency. This phenomenon of ‘withdrawal relief’ provides evidence of an ethanol-induced adaptation which resets the balance of signalling in neural circuits. We have used this as a criterion to distinguish between direct and indirect ethanol-induced adaptive behavioural responses in *C. elegans* with the goal of investigating the genetic basis of ethanol-induced neural plasticity. The paradigm employs a ‘food race assay’ which tests sensorimotor performance of animals acutely and chronically treated with ethanol. We describe a multifaceted *C. elegans* ‘withdrawal syndrome’. One feature, decrease reversal frequency is not relieved by a low dose of ethanol and most likely results from an indirect adaptation to ethanol caused by inhibition of feeding and a food-deprived behavioural state. However another aspect, an aberrant behaviour consisting of spontaneous deep body bends, did show withdrawal relief and therefore we suggest this is the expression of ethanol-induced plasticity. The potassium channel, *slo-1*, which is a candidate ethanol effector in *C. elegans*, is not required for the responses described here. However a mutant deficient in neuropeptides, *egl-3*, is resistant to withdrawal (although it still exhibits acute responses to ethanol). This dependence on neuropeptides does not involve the NPY-like receptor *npr-1*, previously implicated in *C. elegans* ethanol withdrawal. Therefore other neuropeptide pathways mediate this effect. These data resonate with mammalian studies which report involvement of a number of neuropeptides in chronic responses to alcohol including corticotrophin-releasing-factor (CRF), opioids, tachykinins as well as NPY. This suggests an evolutionarily conserved role for neuropeptides in ethanol-induced plasticity and opens the way for a genetic analysis of the effects of alcohol on a simple model system.

## Introduction

Human alcohol dependence is a chronic relapsing disorder characterised by a preoccupation with obtaining alcohol, loss of control over its consumption, tolerance, withdrawal, and impairment in functioning in both social and work related situations [1]. Key factors in the acquisition of human alcohol dependence are the behavioural phenomena of tolerance, withdrawal and withdrawal relief [2]. Currently, the understanding is that these behaviours arise through the ability of ethanol to induce adaptive changes in neural signalling [3], [4]. This is an intricate process as ethanol differentially engages with multiple molecular effectors over a range of ethanol concentrations, from a blood alcohol level of approximately 10 to 50 mM [5]. During ethanol exposure these effector pathways adapt and modify their individual molecular outputs leading to tolerance in which the efficacy of ethanol is reduced. This tolerance may occur within one session of drinking (acute tolerance) or following prolonged, episodic exposure, chronic tolerance [2]. On the other hand, ethanol withdrawal precipitates a behavioural state involving increased anxiety, agitation, tremors and in the most severe cases, seizures [6]. Importantly, this withdrawal syndrome can be relieved by a low dose of ethanol and it has been suggested this is particularly relevant to the establishment of ethanol dependence [4], [7]. The molecular mechanisms underpinning withdrawal are poorly understood, but likely to be complex given the multiple cellular targets through which ethanol can act.

Modelling ethanol-induced behavioural states in genetically tractable animals, including the simple invertebrate models *Drosophila* and *C. elegans*, provides a good opportunity to place multiple and diverse molecular effectors for ethanol in the context of circuits and systems that underpin its pleiotropic behavioural effects. These invertebrates employ the same fundamental neural processes as in higher animals and, for the most part similar neurotransmitter systems, and thus may reveal conserved mechanisms underpinning alcohol dependence [8].

The model genetic invertebrates *Drosophila* and *C. elegans* both show acute and chronic behavioural responses to ethanol exposure [8]–[10]. In terms of relating this to human alcohol dependence, susceptibility to the acute intoxicating effects of ethanol is of interest as there is evidence that acute resistance can predispose an individual to alcohol abuse [11], [12]. In *Drosophila* the acute response is manifest as an initial increase in locomotor activity followed by inhibition [13]. In *C. elegans* too, hyperexcitation has been reported followed by inhibition of locomotor activity and egg-laying [14]–[19]. Adaptive responses to chronic ethanol exposure have also been observed. *C. elegans* shows acute tolerance in which the ethanol effect is reduced during a single session of exposure [17]. *Drosophila* exhibit both rapid and chronic tolerance, in the absence of altered ethanol metabolism, in which the efficacy of ethanol is reduced during consecutive exposures [20], [21].

The observation of ethanol-dependent behaviours in *C. elegans* and *Drosophila* has opened the way for genetic screens. These have identified genetic mutations which confer either hypersensitivity or resistance to ethanol. One of the first mutants isolated in these screens was the *Drosophila cheapdate* mutant, an allele of *amnesiac*, which is hypersensitive to the acute intoxicating actions of ethanol [13]. The gene encodes a neuropeptide that signals through a cAMP-dependent pathway. Further screens have identified a number of genes that confer resistance to the sedative effects of ethanol including involvement of insulin signalling pathways [22], *white rabbit*, which encodes a RhoGAP small molecule G protein [23] and *happyhour* which implicates EGFR signalling [24]. In addition, there are disparate observations concerning the role of a calcium-activated potassium channel, SLO-1. In *C. elegans*, functional null mutants for *slo-1* are reported to be highly resistant to acute ethanol exposure [15]. However, in *Drosophila slo-1* mutants remain sensitive to the acute actions of ethanol although they are defective in functional tolerance [25], [26]. Further studies investigating the genetic basis of the adaptive response to ethanol indicate that this is a complex phenomenon involving multiple gene pathways influenced by octopamine signalling [20] and with a requirement for a nuclear zinc-finger protein encoded by *hangover* [27] in *Drosophila*, whilst in *C. elegans* it has been reported that the development of tolerance is negatively regulated by a neuropeptide Y like receptor NPR-1 suggesting peptidergic signalling contributes to this phenomenon [17]. NPR-1 has also been implicated in withdrawal [17].

An important consideration in the interpretation of studies investigating behavioural responses to chronic ethanol exposure is whether or not the observed effects are a manifestation of a direct ethanol-induced neuroadaptation or whether they are due to an indirect effect, for example as a consequence of ethanol as a non-specific environmental stressor. A recent paper reported that chronic exposure of *C. elegans* to ethanol resulted in broad-ranging effects on development, fecundity and feeding [28]. Any of these chronic effects of ethanol may cause behavioural changes that persist after ethanol removal and could thus confound the analysis of ethanol withdrawal.

Here we have used a behavioural paradigm requiring sensorimotor coordination, navigation towards a food source, to show that *C. elegans* acutely exposed to ethanol exhibit behaviour that is distinct from worms chronically exposed to, and subsequently removed from ethanol. Intriguingly the latter undergo a multi-faceted behavioural adaptation only one component of which is relieved by a low dose of ethanol. This component is an aberrant behaviour consisting of an increased frequency of spontaneous body bends. We have used this paradigm to investigate the genetic basis for ethanol withdrawal and show that the NPY-like receptor, NPR-1, does not affect withdrawal in this assay suggesting other genes are involved. Whilst NPY-like peptides signalling through NPR-1 are not involved, the evidence provided here show that neuropeptides do play a pivotal role as a mutant defective in neuropeptide synthesis does not show withdrawal. This resonates with data emerging from similar investigations in mammalian systems which suggest that neuropeptides are important mediators of neuroplasiticity underpinning alcoholism [29]–[34].

## Results

### A navigational task, the ‘food race’ provides a read-out of ethanol-induced behaviour

**Figure 1:**
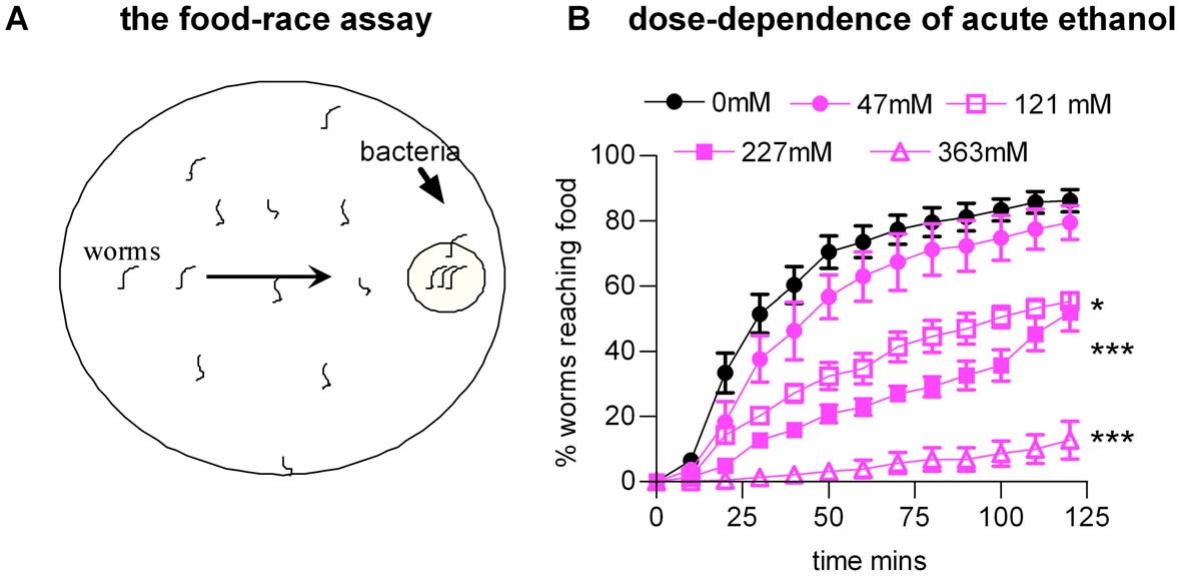
Acute exposure to ethanol impairs performance in a food race assay. **A** Diagram to represent the food-race paradigm. Each race was performed for a population of worms (approximately 50 per race) on 9 cm agar plates with a single spot of *E. coli* 2 cm from the edge of the plate. At time zero, staged worms (L4 plus 1 day) were aliquoted on to the plate diametrically opposite the food source. Every 10 minutes, the number of worms that had reached the food was counted and expressed as a % of the total population. Data are expressed as the mean ± s.e.mean of ‘n’ experiments where each experiment is a single food race. **B** The effect of performing the race in the presence of ethanol. Ethanol was added to the agar to give an approximate concentration of 50, 100, 250 and 400 mM. Plates were tested for ethanol concentration after use and the average concentration is indicated on the graph. 0 mM (n = 11); 47 mM (n = 4, p>0.05 compared to 0 mM); 100 mM (n = 5; p<0.05 compared to 0 mM); 227 mM (n = 4; p<0.001 compared to 0 mM); 363 mM (n = 4; p<0.001 compared to 0 mM). Statistical analysis was performed using one way ANOVA with Bonferroni multiple comparisons post-test on the last time-points.

Previous work indicates a dose-dependent (100 to 500 mM) effect of ethanol on *C. elegans* locomotory behaviour [15], [17], [18], [19], [35]. However, we reasoned that some aspects of ethanol's effects might better be revealed by subjecting the worms to a more complex behavioural task that relies on sensorimotor processing e.g. foraging. This can be modelled simply and quantitatively in a ‘food race’ assay. In this assay approximately 50 worms are added to the opposite side of the plate to a point source of food, E. coli OP50 (Figure 1A). Over time the worms navigate towards the food and their efficiency of navigation can be quantified by counting the percentage of worms that reach the food in a given time. Ethanol at concentrations of 121 mM, 227 mM and 363 mM reduced the ability of worms to reach the food whilst 47 mM had no significant effect (Figure 1B). A previous study has provided evidence that ethanol rapidly equilibrates across the worm cuticle and therefore this external concentration is likely to be a close approximation of the tissue concentration [18].

### Chronic exposure to ethanol prior to the food race impairs navigation: A withdrawal state

**Figure 2:**
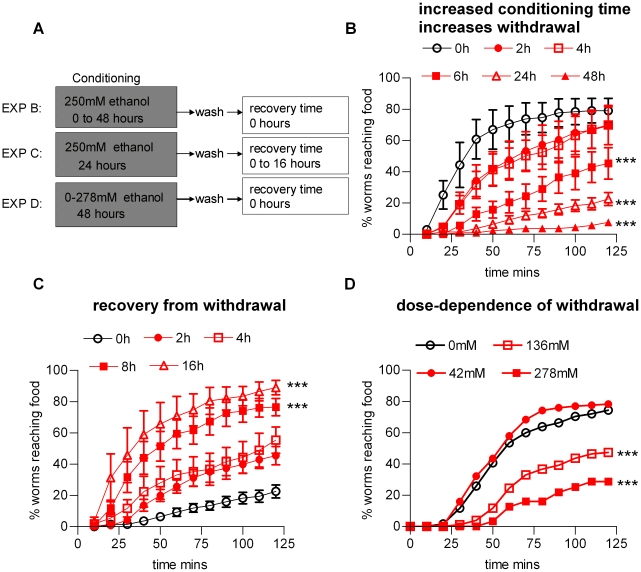
Ethanol conditioning induces a withdrawal phenomenon in the food race. **A** Diagram showing the conditioning paradigm. Developmentally staged worms were placed on agar plates with an excess of E. coli OP50 and agar containing ethanol. At the end of the conditioning period, the worms were washed from the plates in M9 buffer, washed once more in M9 to remove residual ethanol and E. coli, and then aliquoted onto the food race plate. Three sets of experiments were performed in which either the conditioning time was varied (B), or the conditioning time was set and the recovery time was varied (C), or the conditioning time and recovery time were set and the conditioning concentration of ethanol was varied (D). Parallel controls were performed in which ethanol naive worms raised for similar time-periods were tested for performance in the food race. These indicated no significant effect of the culture time on performance (95±5%, n = 3, reached the food source by the 2 h time-point for L4 plus 1 day worms, and 93±4% for L4 plus 2 days.) **B** The effect of varying the time-course of ethanol conditioning on performance in the food race. Worms were conditioned on ethanol for the time indicated. 0 h (n = 5); 2 h (n = 6; p>0.05 compared to 0 h); 4 h (n = 4; p>0.05 compared to 0 h); 6 h (n = 7; p<0.001 compared to 0 h); 24 h (n = 6; p<0.001 compared to 0 h); 48 h (n = 4; p<0.001 compared to 0 h). **C** The effect of varying the recovery time from conditioning on performance in the food race. The recovery times are as indicated. 0 h (n = 6); 2 h (n = 4; p>0.05 compared to 0 h); 4 h (n = 4; p>0.05 compared to 0 h); 8 h (n = 4; p<0.001 compared to 0 h); 16 h (n = 4; p<0.001 compared to 0 h). The experiment was also performed on worms conditioned on ethanol for 48 h which exhibited similar recovery times (see text for details). **D** The effect of varying the conditioning ethanol concentration on performance in the food race. Worms were conditioned on ethanol for 48 h at the concentration indicated. Each point is the mean of a duplicate determination. Statistical analysis was performed using one way ANOVA with Bonferroni multiple comparisons post-test on the last time-points.

*C. elegans* chronically exposed to ethanol, for 6 h up to 48 h, and then tested in the food race in the absence of ethanol, also exhibited impaired performance in the food race. For these assays worms were preconditioned, either in the presence or absence of 250 mM ethanol and then tested in the food race (Figure 2A). The data in Figure 2B show the effect of conditioning the worms in ethanol for different time periods and then measuring their performance in the food race assay immediately after they were removed from the ethanol plates and washed. This shows that the longer the worms were exposed to ethanol prior to removal, the greater the withdrawal effect and furthermore that at least 6 h exposure to ethanol was required to induce this effect. Worms could recover from this withdrawal state if left for sufficient time in the absence of ethanol (Figure 2C). The longest conditioning time tested was 48 h. Withdrawal from this prolonged conditioning time resulted in just 9±5% of withdrawn worms reaching the food. However this effect was reversible as after 24 h recovery off ethanol this improved to 65±11% (n = 4; p<0.05). The lowest concentration at which ethanol-induced withdrawal occurred was between 42 and 136 mM ethanol (Figure 2D) and thus occurred over a similar concentration range as the acute ethanol effects.

### A low dose of ethanol improves the performance of worms experiencing ethanol withdrawal

**Figure 3:**
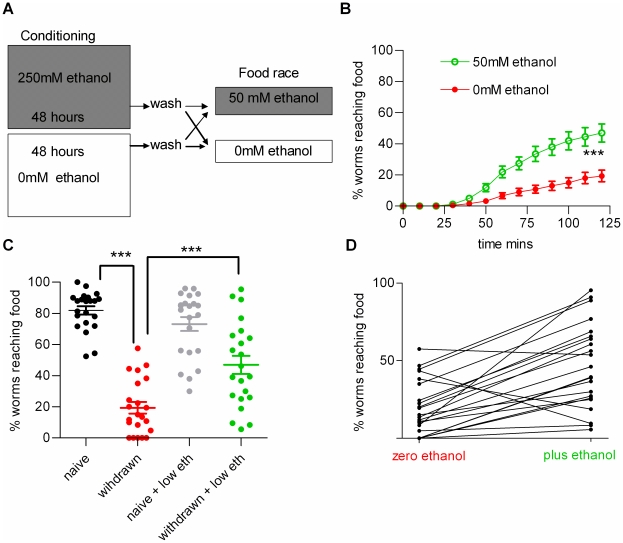
Withdrawal can be relieved by a low dose of ethanol. **A** Diagram to show the paradigm used to demonstrate relief from withdrawal. Developmentally staged worms (L4 plus 1 day) were conditioned on abundant food in the presence of 250 mM ethanol for 48 h, recovered from the plates by washing and then tested in the food race either in the absence or presence of ethanol (added to the plates to give an approximate final concentration of 50 mM, mean measured value was 66±2 mM, n>50). **B** The time-course of the effect of 66±2 mM ethanol on conditioned worms (n = 22; there is a significant effect of ethanol treatment, p<0.001; two way ANOVA). **C** A comparison for all 4 experimental groups described in A, at the 2 h end time-point of the food race. Each point is the value obtained from a single food race experiment. The bars indicate the mean ± s.e.mean. ‘naive’ have not been pre-exposed to ethanol and are tested in the absence of ethanol; ‘withdrawn’ have been pre-exposed to ethanol and are tested in the absence of ethanol; ‘naive + low eth’ have not been pre-exposed to ethanol and are tested in the presence of a low dose of ethanol; ‘withdrawn + low eth’ have been pre-exposed to ethanol and are tested in the presence of a low dose of ethanol. Statistical analysis was performed using one way ANOVA with Bonferroni multiple comparisons post-test. *** p<0.001. **D** A comparison of all the paired data sets for conditioned worms tested in the absence (zero) and presence of 66±2 mM ethanol (plus ethanol). The lines connect data from paired groups. 4 out of 22 groups did not show withdrawal relief.

**Figure 4:**
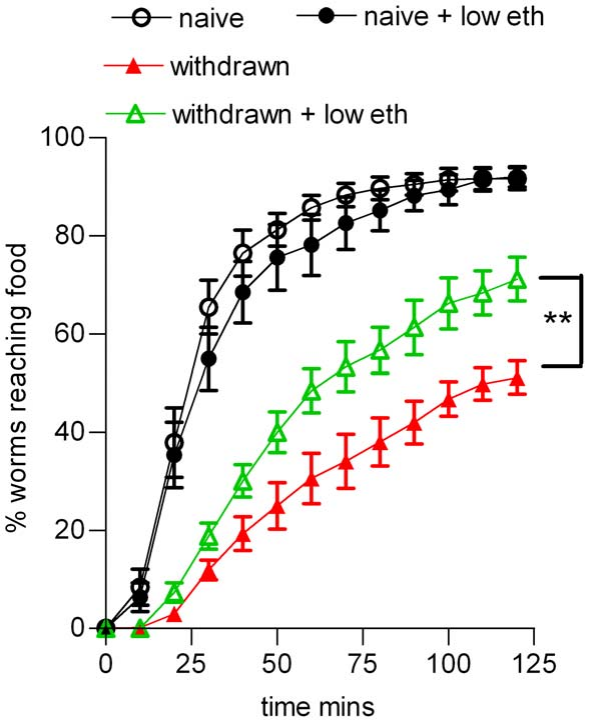
Withdrawal and withdrawal relief can be observed at shorter periods of ethanol conditioning. Developmentally staged worms (L4 plus 1 day) were conditioned on abundant food in the absence (naive) or presence (withdrawn) of 354±32 mM ethanol for 6 h, recovered from the plates by washing and then tested in the food race either in the absence (naive, withdrawn) or presence (60±1 mM; naive + low eth, withdrawn + low eth) of ethanol. Each point is the mean ± s.e.mean of 4 independent determinations for 50 worms in a food race. Statistical analysis was performed using one way ANOVA with Bonferroni multiple comparisons post-test on the last time-points. ** p<0.01.

Evidence that withdrawal behaviour does not arise from an ethanol-induced developmental defect or irreversible toxicity is provided by the observation that the effect was reversible (see above) and that a low dose of ethanol relieved the withdrawal behaviour. For these experiments 50 mM ethanol, which in itself did not inhibit performance in the food race (Figure 1B), was present in the agar plate on which the food races were conducted for both the naive and ethanol withdrawn worms (Figure 3A). This exposure to a low dose of ethanol significantly improved the performance of worms experiencing ethanol withdrawal (Figure 3B, 3C). This phenomenon has the properties of ‘withdrawal relief’ and occurred in 18 out of 22 populations studied (Figure 3D). The reason for the incomplete penetrance of this ethanol behaviour in wild-type worms probably results from the pleiotropic actions of ethanol such that the net behaviour of the population results from a fine balance between many different and possibly opposing actions of ethanol exposure [28]. Nonetheless, withdrawal relief in the majority of populations studied indicates that the inability of ethanol withdrawn worms to reach the food is unlikely to be due to ethanol inducing a satiated state, nor with it impairing the ability of the conditioned worms to detect the food source, nor with it persisting in the worm following the conditioning protocol. (This latter point is further supported by earlier evidence indicating ethanol rapidly diffuses across the cuticle, 18, therefore the wash procedure after conditioning will effectively remove residual ethanol). Withdrawal and withdrawal relief was also observed at a shorter conditioning time of 6 h (Figure 4). Overall, this supports the contention that withdrawal and withdrawal relief are indicators of ethanol-induced plasticity in *C. elegans*.

### Acute and chronic effects of ethanol are distinct behavioural states

**Figure 5:**
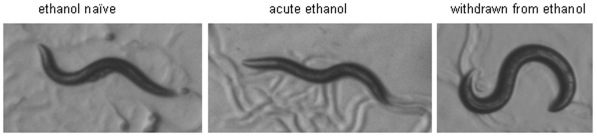
The body posture of worms during acute exposure to ethanol and during ethanol withdrawal is different. The image of the left is a wild-type ethanol naive worm in the absence of ethanol, in the middle is an image of a worm in the presence of 250 mM ethanol, and on the right following exposure to and withdrawal from 250 mM ethanol.

**Figure 6:**
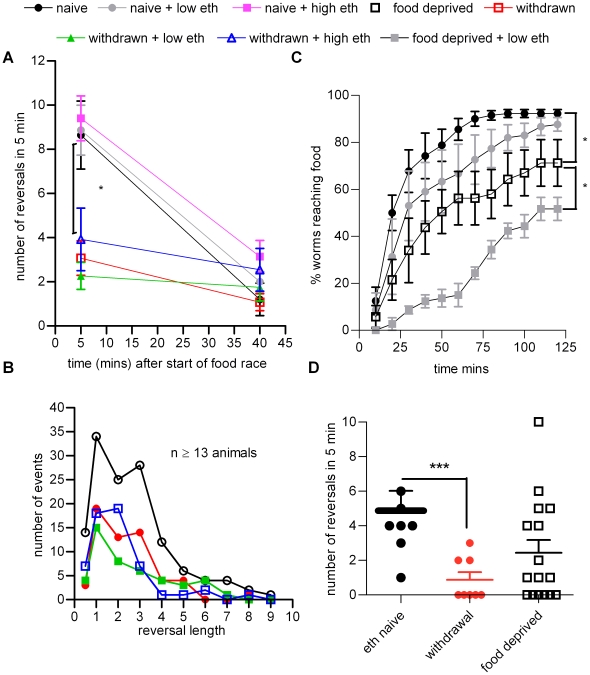
The effect of ethanol on the frequency of reversals. **A** In order to quantify the effect of ethanol conditioning on locomotor activity, the conditioning experiments were repeated using the same protocol as shown in Figure 2A except that a 6 h conditioning time was adopted and video recordings were made. Reversals were scored for 5 min for each animal between 5–10 and 40–45 min into the food race. For these and all subsequent figures the same colour code is used to depict each of the different ethanol treatment groups. Statistical analysis was performed using one-way ANOVA with Bonferroni multiple comparisons between the different treatment groups for each time-point. No statistical significance between groups was observed at 40 min. At 5 min statistical differences were observed between all the ethanol naive groups compared to the ethanol withdrawn groups as indicated. Data are presented as mean ± s.e.mean. For the 5 min time-point n = 13 to 15. For the 40 min time-point n = 5 to 15. **B** Ethanol conditioning did not affect length of reversals. The length of reversal was measured by counting the number of head turns the worm made during the backward movement, where one head turn occurs for one reversal length. Data collected from 5 min recordings of at least 13 worms per condition, taken 5 min after being placed on a food race plate. Note that the proportion of reversals of a given length is not different between naive and conditioned animals, even though the number of reversals is reduced. C. Food deprivation impairs navigational performance and sensitizes worms to ethanol. Wild-type worms (developmentally staged to L4+1 day) were picked onto plates either in the presence or absence of food. Following 6 h they were recovered from the plates by washing in M9 and then tested in a food race with or without the addition of (66±4 mM) ethanol. Statistical significance was tested using one way ANOVA with Bonferroni's multiple comparison post-test on the last time-points (n = 3 to 4). **D** The effect of food deprivation on reversals. Wild-type worms (developmentally staged to L4+1 day) were conditioned on (231±9 mM) ethanol or incubated in the absence of food for 6 h (±15 min). A control group was also incubated with abundant food for 6 h. Following this, individual worms were recovered by collecting in M9 and transferred to a food race plate. 5 min into the race, worms were video recorded for 5 min for later analysis. Manual analysis of each video was carried out and the number of reversals was counted for each worm. Each data point is a value from one worm and the bars indicate mean ± s.e.mean. Statistical significance was determined with one way ANOVA and Bonferroni's multiple comparison post-test.

The behaviour and appearance of worms acutely exposed to ethanol compared to those in ethanol withdrawal was distinctive (Figure 5) despite the fact that the net read-out in the food race assay was the same i.e. impaired navigation towards the food. Thus, whilst the former show a slightly shallow waveform, uncoordinated body bends and an inability to move efficiently, in agreement with previous descriptions [15], worms undergoing withdrawal had a distinctly different locomotor pattern consisting of many spontaneous deep body bends and turns (Videos S1, S2 and S3). This suggested that chronic ethanol exposure impacted on the normal *C. elegans* strategy for finding food which involves coupling reversals and high-angled turns to facilitate re-orientation and navigation [36], [37]. Therefore we sampled the frequency of both these behaviours, reversals and high-angled turns at an early (5 min) and late (40 min) time-point during the food race, for ethanol naive and conditioned worms, and in the absence and presence of ethanol. In terms of reversals, the different ethanol treatment groups fell into two distinct categories (Figure 6A). Naive worms (that had never been exposed to ethanol i.e. naive) and worms that had been acutely exposed to ethanol (either a low or high dose) all behaved in the same manner with a high frequency of reversals at the 5 min time-point which decreased to a lower value at the 40 min time-point. In contrast, all the ethanol treatment groups that were pre-treated by chronic exposure on ethanol (withdrawn) had a much lower frequency of reversals at the 5 min time-point which remained at the low level after 40 min. Whilst reversals in these withdrawn treatment groups were at a lower frequency compared to unconditioned worms, reversals were not of an altered length (Figure 6B). It is quite striking that the acutely ethanol treated worms, for which navigational behaviour is severely impaired (Figure 1B) still show the same reversal frequency as ethanol naive worms (Figure 6A).

We considered whether the decrease in reversal frequency observed in the ethanol withdrawn treatment groups was indicative of a neuroadaptation to ethanol. However, this behaviour was not relieved by a low dose of ethanol (Figure 6A). We speculated that the effect on reversal may not be a direct consequence of ethanol-induced plasticity but rather an indirect behavioural adaptation to one of the pleiotropic actions of ethanol [28] e.g. inhibition of feeding [18], [28]. To test this we compared the performance of worms that had been subjected to food-deprivation with those that were in ethanol withdrawal. We found that food-deprived worms also performed poorly in the food race (Figure 6C). This is consistent with a reported reduction in reversal frequency following starvation [38] and we also found that food-deprived worms showed a trend towards a reduction in reversal compared to well-fed worms in the food race (Figure 6D; p = 0.07). A reduction in reversal frequency would provide an explanation for impaired navigation [37]. Taken together these data indicate that one effect of chronic ethanol is to induce an indirect behavioural adaptation to food deprivation, most likely through its ability to inhibit pharyngeal pumping. Importantly, there is a clear difference between the effect of a low dose of ethanol on food-deprived, compared to ethanol withdrawn worms, in that the performance of food-deprived worms in the food race is further impaired, not improved, by a low dose of ethanol (Figure 6C). The diametrically opposed effects of a low dose of ethanol on worms in ethanol withdrawal, compared to food withdrawal, indicate that these two behavioural states are distinct.

**Figure 7:**
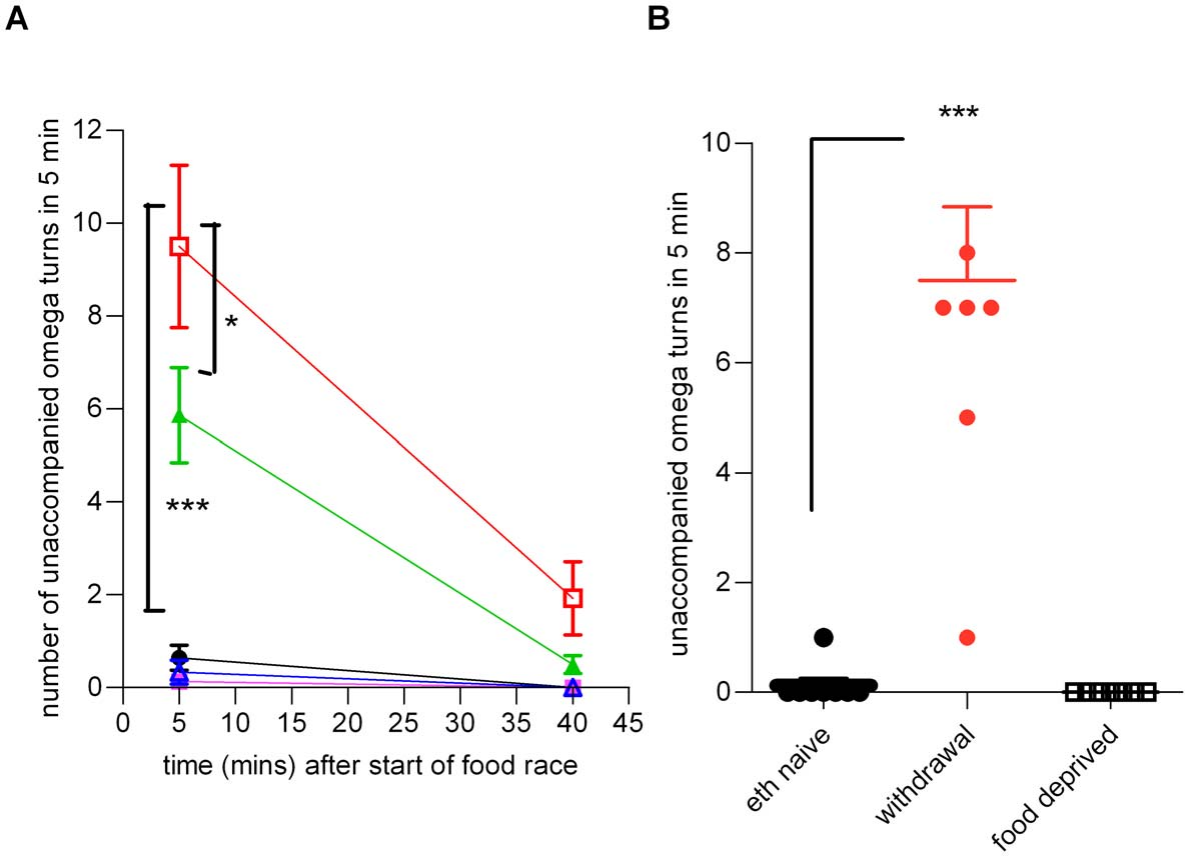
Ethanol withdrawal, but not food deprivation, increases the frequency of unaccompanied omega turns. **A** The conditioning experiments were repeated using the same protocol as shown in Figure 2A except that a 6 h conditioning time was adopted and video recordings were made. Unaccompanied omega turns were scored for a 5 min period for each animal between 5–10 and 40–45 min into the food race. Statistical analysis was performed using one-way ANOVA with Bonferroni multiple comparisons between the different treatment groups for each time-point. No statistical significance between groups was observed at 40 min. At 5 min statistical differences were observed as indicated. Data are presented as mean ± s.e.mean. For the 5 min time-point n = 13 to 15. For the 40 min time-point n = 5 to 15. * p<0.05, *** p<0.001. **B** Wild-type worms (developmentally staged to L4+1 day) were conditioned on (231±9 mM) ethanol or incubated in the absence of food for 6 h (*±15 min). A control group (eth naive) was also incubated with abundant food for 6 h. Following this, individual worms were recovered by collecting in M9 and transferred to a food race plate. 5 min into the race, worms were video recorded for 5 min for later analysis. Manual analysis of each video was carried out and the number of omega turns which were not preceded by a reversal was counted for each worm. Each data point is a value from one worm and the bars indicate mean ± s.e.mean. Statistical significance was determined with one way ANOVA and Bonferroni's multiple comparison post-test. *** p<0.001.

The second aspect of behaviour relevant to navigation that we quantified was high angled turns, called omega turns. These commonly occur after a reversal and facilitate a change in direction and only rarely occur independently of reversals [37]. Ethanol withdrawal resulted in a striking and significant increase in the percentage of omega turns that occurred independent of a reversal (‘unaccompanied’ omega turn) at the 5 min time-point in the food race (Video S3 shows examples of unaccompanied omega turns; The unaccompanied omega turns were 10% of the total omega turns in ethanol naive worms but 87% of the total in worms experiencing ethanol withdrawal). The absolute frequency of ‘unaccompanied’ omega turns was significantly increased in ethanol withdrawal at the 5 min time-point in the food race (Figure 7A). This effect, of increased unaccompanied omega turns, was only observed in ethanol withdrawal and not in food withdrawal (n = 8; Figure 7B). Furthermore, the increase in unaccompanied omega turns was relieved by 50 mM ethanol (Figure 7A), a low dose which in itself did not affect locomotor performance (Figure 1B). Thus the appearance of unaccompanied omega turns in ethanol withdrawal appears to be a direct and specific effect of the ability of ethanol to alter the properties of signalling in a neural network, and not an indirect effect of ethanol inhibition of feeding. The increase in frequency of unaccompanied omega turns decreased over time in the food race. After 40 min on the food race plate there was little difference compared to ethanol naive worms. This is unlikely to reflect recovery from withdrawal which occurs over a longer time-course (Figure 2C), but may reflect temporal regulation of the behaviour of the worms on the food race plate, and perhaps also spatial regulation as they migrated nearer to the food source. As the ethanol withdrawal behaviour of increased unaccompanied omega bends was only observed at the early, 5 min, time-point in the food race it is clearly a highly plastic phenomenon.

Withdrawn worms also had a much greater tendency to curl into a ball after a reversal compared to those acutely exposed to ethanol (% of reversals that were followed by curling up into a ball were, naive, 2%; acute 250 mM ethanol, 5%; withdrawn, 24%; n≥100 reversals on ≥10 worms; Video S4). This behaviour also showed ethanol withdrawal relief as withdrawn worms placed on 50 mM ethanol showed a marked amelioration of this behaviour with only 3% of reversals leading to the worm curling up into a ball.

The data above can be summarised as follows: Ethanol conditioning induced two behavioural states that can be distinguished in animals in the first 5 min in a food race task. There was a decrease in reversal frequency that was not relieved by a low dose of ethanol and may, at least in part, be explained by an adaptation to food deprivation. Secondly, there was an increase in the frequency of unaccompanied omega turns. This behaviour was only seen following ethanol conditioning and was not caused by food deprivation. It contrasts markedly with the behaviour of ethanol naive animals in which reversals and omega turns were typically coupled in a pattern of movement termed ‘pirouettes’ associated with local area search behaviour [36], [37]. This increase in unaccompanied omega turns was ameliorated by a low dose of ethanol from which we deduce that it is an adaptive behaviour to chronic ethanol exposure.

### The role of SLO-1 and NPR-1 in the effect of ethanol on navigational performance

Previous studies in *C. elegans* have implicated two signalling pathways in the acute and chronic effects of ethanol. Therefore we tested whether mutants for either of these pathways had altered responses to either acute or chronic ethanol in the food race assay.

**Figure 8:**
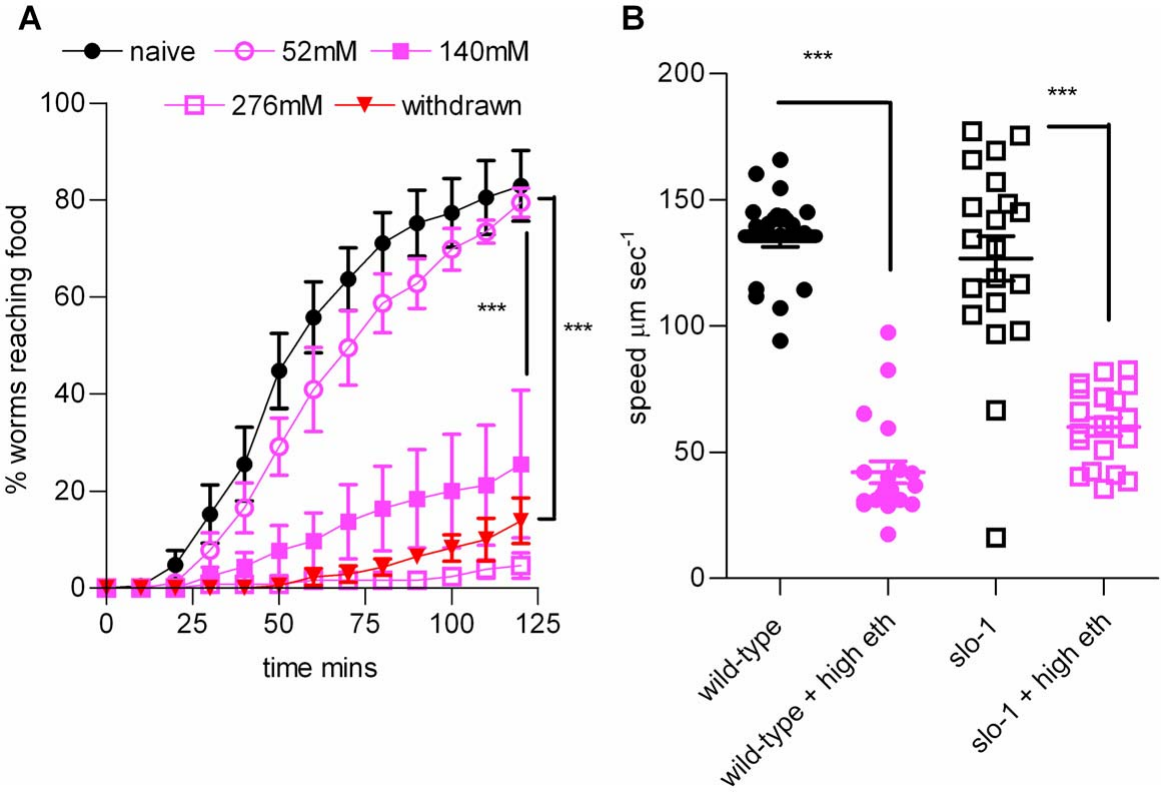
The effect of acute and chronic exposure on performance in the food race for the BK channel mutant, *slo-1(js379)*. The food race assays were performed as described for wild-type worms (described in legend to Figure 1). **A** The performance of *slo-1(js379)* worms in the food race in the presence of increasing concentrations of ethanol as indicated. Each point is the mean ± s.e. mean of 4 to 6 determinations. *** p<0.001 one-way ANOVA with Bonferroni multiple comparisons post-test on the last time-points. **B** Comparison of the effect of acute ethanol (250 mM) on worm locomotory speed for wild-type and *slo-1(js379)*. Speed was measured as described in the legend for Figure 10A. Each data point represents a measurement from a single worm and the bars indicate the mean ± s.e. mean for each data set. *** p<0.001, one way ANOVA with Bonferroni multiple comparisons post-test.

The gene *slo-1* encodes the main pore forming subunit of the BK potassium channel, and worms with loss of function mutations in this gene were identified in a screen for worms resistant to the acute effects of ethanol [15]. However, in the food race assay *slo-1 (js379)*, which are predicted to be a functional null for the channel [39] showed a similar navigational impairment to wild-type animals in the presence of ethanol (Figure 8A) and reduction in locomotory speed on food race plates (Figure 8B). In a further series of experiments we tested the sensitivity of two additional *slo-1* mutants, *pd24* and *pd23*, both predicted to be reduction or loss of function mutants [40], and observed a similar sensitivity to acute ethanol exposure as seen in wild-type (23±5% of *pd23* and 7±3% of *pd24* mutants reached the food after 2 h on 200 mM ethanol). The mutant *slo-1(js379)* also exhibited ethanol withdrawal in a similar manner to wild-type animals (Figure 8A). Therefore the food race assay is unable to distinguish an influence of *slo-1* dependent signalling on acute or chronic responses to ethanol.

**Figure 9:**
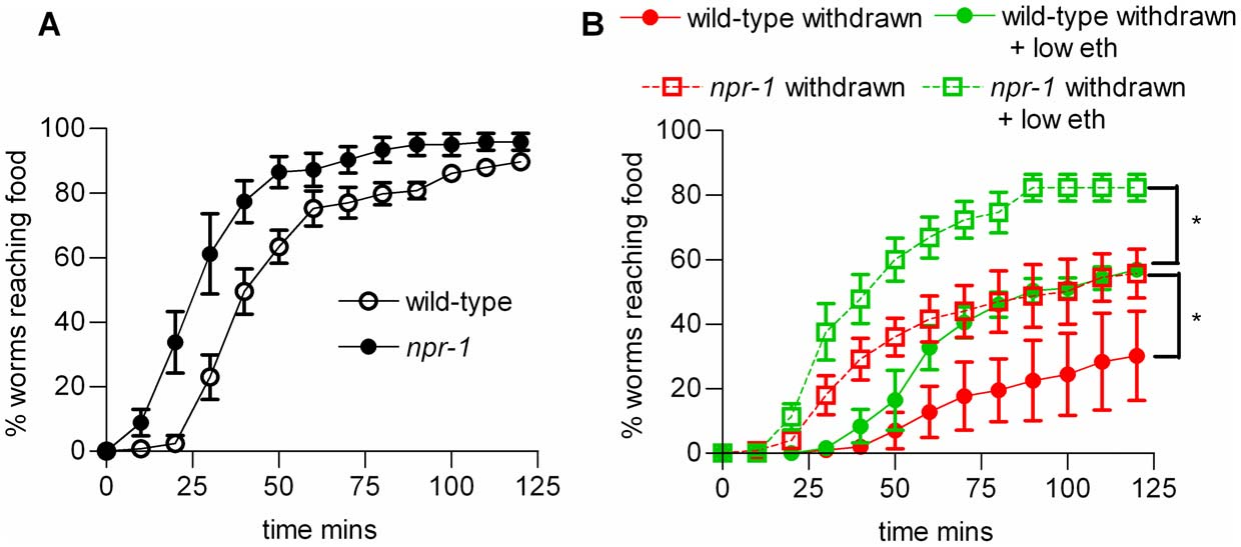
The effect of acute and chronic exposure on performance in the food race for the NPY-like receptor null mutant, *npr-1(ky13)*. The food race assays were performed as described for wild-type worms (described in legend to Figure 1). **A** The performance of *npr-1(ky13)* worms in the food race compared to wild-type. Both groups of worms were ethanol naive and tested in the absence of ethanol. Each point is the mean ± s.e. mean of 3 to 4 determinations. **B** The effect of ethanol conditioning on *npr-1(ky13)* compared to wild-type. Worms were conditioned for 48 h on 180 mM ethanol, removed and tested in the food race in the absence or presence of 70 mM ethanol. Each point is the mean ± s.e.mean of 3 to 4 determinations. * p<0.05 using one-way ANOVA with Bonferroni multiple comparisons post-test on the last time-points.

In *C. elegans* it has been reported that worms with a reduction of function of an NPY-like receptor, NPR-1, phenocopy ethanol withdrawal [17]. This is of interest as many different peptides have been implicated in chronic responses to ethanol in mammals including NPY [41], CRF [30] and the opioid peptides [42], [43]. In *C. elegans*, NPY-like signalling has been implicated through observations of the behaviour of worms when feeding on a bacterial lawn. Both *npr-1* ethanol naive mutants [44], and wild-type worms experiencing ethanol withdrawal [17], accumulate at the edge of the lawn and feed in groups, behaviours called bordering and clumping, respectively. Thus it was suggested that *npr-1* mutants phenocopy ethanol withdrawal and that in withdrawal there is a reduction in *npr-1* signalling. On this basis it would be predicted that *npr-1* (*ky13*), a putative null, would also phenocopy ethanol withdrawal in the food race task and reach the food more slowly than wild-type worms. However, we observed that *npr-1* reached the food at least as efficiently as wild-type (Figure 9A) and therefore, in the food race assay *npr-1* (*ky13)* does not phenocopy ethanol withdrawal. *npr-1* also responded to ethanol conditioning in a similar manner to wild-type worms (Figure 9B). Thus *npr-1* does not affect the response to ethanol conditioning as measured in this navigational task. As ethanol inhibits pharyngeal pumping [18], [28], it is possible that the bordering behaviour observed in wild-type worms in ethanol withdrawal, which is apparently similar to the behaviour of *npr-1* [17], is an adaptive response to food deprivation rather than a direct neuroadaptive response to ethanol.

### A quantitative analysis of ethanol-induced behavioural states in the food race

We further characterised withdrawal behaviour by employing an automated analysis of the behaviour of worms in the 5 min of the food race. This time-point was chosen as the *C. elegans* withdrawal syndrome was observed early in the food race task. The approach employed in-house software based on a Gaussian Mixture Model (see methods and Figure S1 for detail) that provided a measure of speed and two additional parameters that describe navigational behaviour on the food race plates, a ‘posture measure’ that quantifies bendiness and ‘efficiency’ which is defined by dividing the distance traveled by the centre of mass of the worm by the distance of the sinusoidal path that the worm traversed (Figure S1C,D).

**Figure 10:**
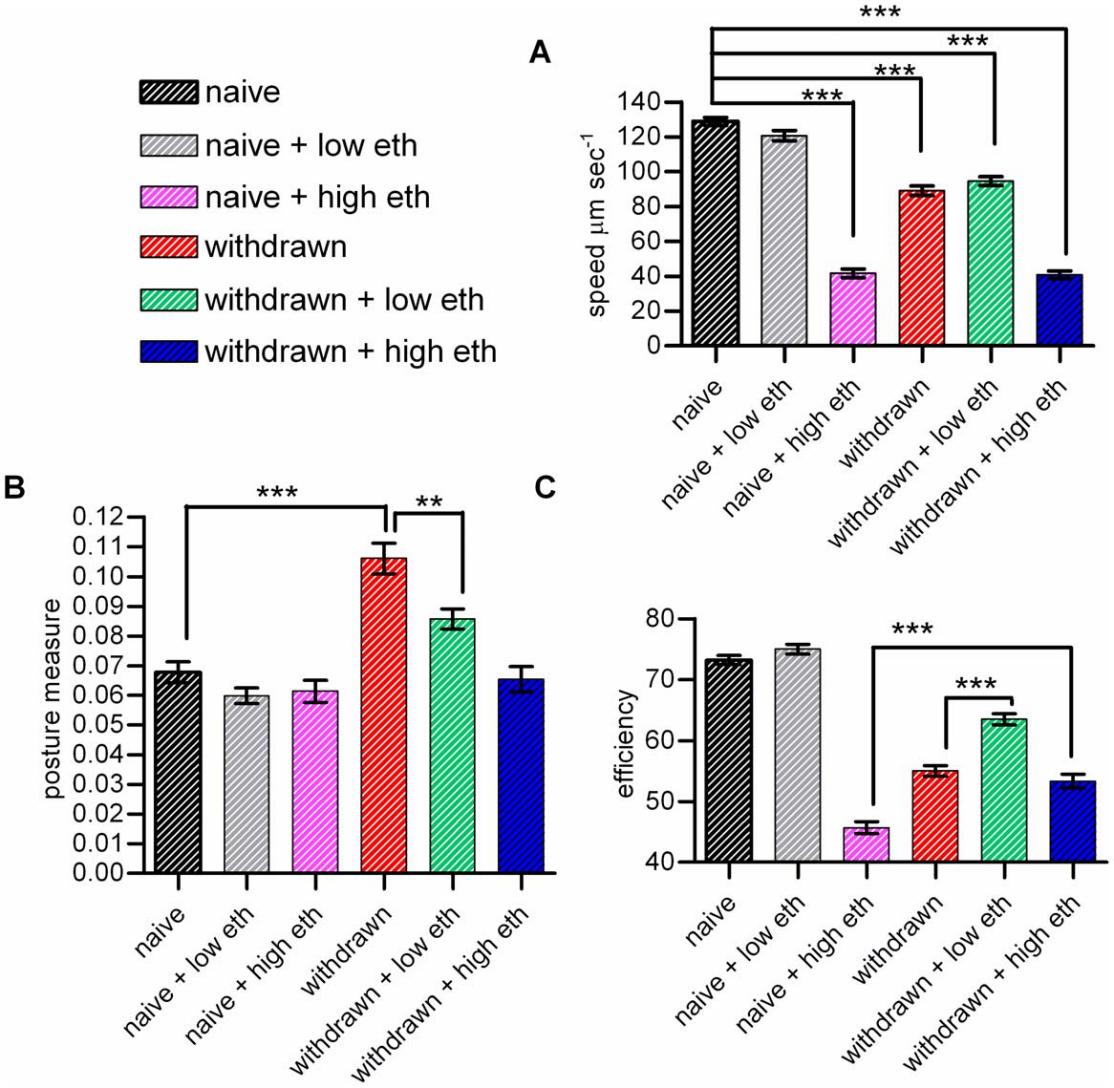
Automated analysis of locomotory behaviour in worms acutely exposed to, or withdrawn from, ethanol confirms distinctive ethanol-induced behavioural states. **A, B and C.** Worms were recorded in the food race in different ethanol treatment groups as described in the legend to Figure 6. Three measurements of motility were made from 30 s videos captured after 5 min on the food race plate, using in house software to determine **A** ‘speed’, **B** ‘posture measure’ which provides a read out of the bendiness of the worm and **C** ‘efficiency’ which provides an indication of the translation of the overall movement of the animal into its trajectory. n = 19 to 22 assays of 20 worms per assay. For **A**, Bonferonni post-tests were performed for all treatment groups compared to naive. For **B** and **C** relevant post-tests with significance are indicated. ** p<0.01, *** p<0.001.

Acute (high) and chronic ethanol exposure both reduced locomotory speed (Figure 10A). Withdrawn worms also showed an increased posture measure, consistent with the observation (described earlier in this paper) that these worms have increased omega turns and tendency to coil up into a ball (Figure 10B). This increase in posture measure was reduced in 50 mM ethanol consistent with the withdrawal relief effect observed for unaccompanied omega turns (Figure 7B, 10B). Worms acutely or chronically exposed to 250 mM ethanol also showed a reduced efficiency of movement mirroring their poor performance in the food race (Figure 10C). Ethanol withdrawal was accompanied by ethanol tolerance, as efficiency was significantly improved in those worms that had been pre-conditioned on ethanol before exposure to a high dose of ethanol compared to those that were exposed to a high dose of ethanol but with no pre-conditioning treatment (Figure 10C).

### Peptidergic signalling is required for ethanol withdrawal

Although the NPY-like receptor *npr-1*, previously implicated in ethanol tolerance and withdrawal [17], does not appear to be involved in the acute and adaptive behavioural responses to ethanol described here, there are many other neuropeptides and neuropeptide receptors in *C. elegans* [45], [46], [47], [48] any of which could contribute to ethanol plasticity. Therefore, to test whether or not peptidergic signalling is involved in *C. eleg*ans ethanol responses we adopted a more expansive approach and investigated the behaviour of a mutant for *egl-3* which encodes a *C. elegans* homologue of a mammalian proprotein convertase [49] and is deficient in the processing of seventy-eight out of seventy-nine neuropeptides detected by MALDI-TOF analysis [50].

**Figure 11:**
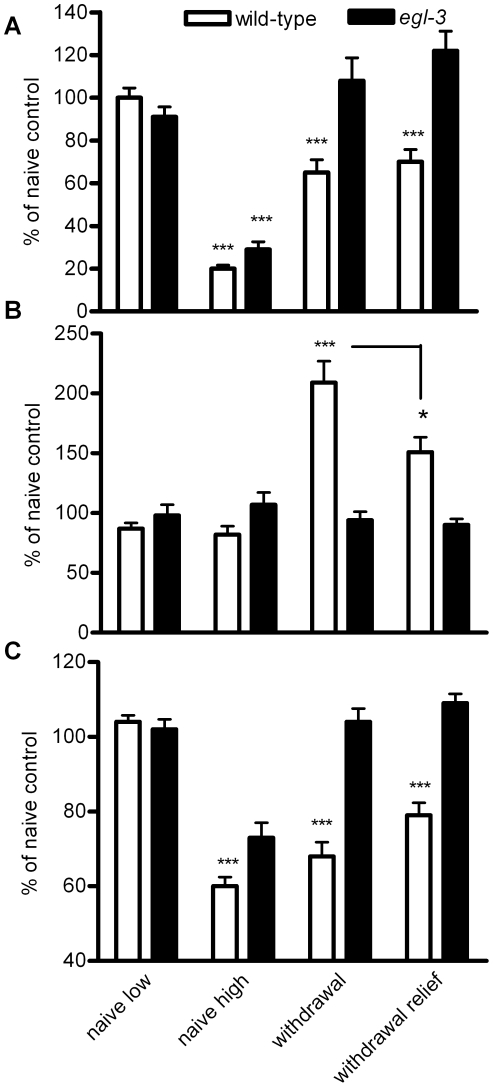
A mutant deficient in peptidergic signalling, *egl-3 (ok979)* exhibits acute but not chronic responses to ethanol. Data were collected and analysed as described in Figure 10. n = 19 to 20 worms for wild-type and *egl-3*. Data have been normalised to the ethanol naive state for each strain and are represented as % control. **A** Speed **B** Posture measure **C** Efficiency. There was a significant interaction between genotype and ethanol treatment for all three measurements (speed, F = 9.17, p<0.0001; posture, F = 12.24, p<0.0001; efficiency, F = 13.73, p<0.0001). (Significance shown is between ethanol naive and the ethanol treatment for each genotype; except for posture measure for which the significance shown is between ethanol naive and ethanol withdrawal for wild-type, and ethanol withdrawal and withdrawal relief, also for wild-type, *** p<0.001, * p<0.05).

**Figure 12:**
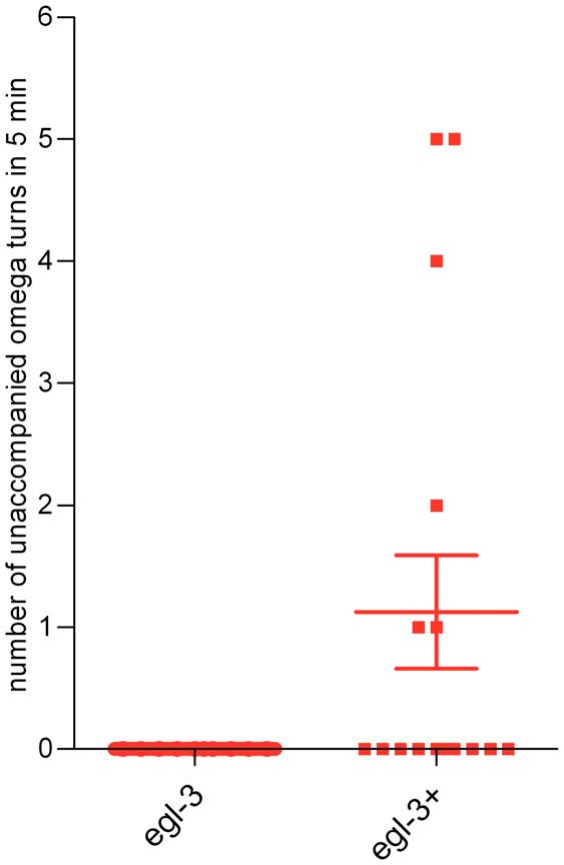
The ethanol withdrawal phenotype of increased unaccompanied omega turns was rescued by expression of wild-type *egl-3* in *egl-3(ok979)*. The mutants were transformed with cosmid harbouring a wild-type copy of *egl-3* behind the native *egl-3* promoter. Independent transgenic lines or *egl-3* control (transformed with the transformation marker only, *Pmyo-2::gfp*) were subject to ethanol conditioning (6 h, 250 mM) and assayed for unaccompanied omega turns (as described in Fig 3B). The data points are the value obtained from single worms. n = 10 to 16. Rescue was observed in 4 independent lines.

Baseline measures of *egl-3* mutants indicated that the speed and posture measure of ethanol naive *egl-3(ok979)* was not significantly different from ethanol naive wild-type worms (129±7 µm sec^−1^ for wild-type compared to 118±5 µm sec^−1^ for *egl-3(ok979)*; n = 18 to 20, p>0.05; for posture measure, 0.056±0.003 for wild-type compared to 0.075±0.003 for *egl-3*; p>0.05; n = 18 to 20). The response of *egl-3* to acute 250 mM ethanol exposure was similar to wild-type with a reduction in speed, but *egl-3* in ethanol withdrawal responded differently (Figure 11). They did not show a reduction in speed (Figure 11A) and, most strikingly, did not show an increase in posture measure during ethanol withdrawal (Figure 11B), nor an increase in the frequency of unaccompanied omega turns (Figure 12). This was paralleled by a higher efficiency of movement (Figure 11C). Overall, although *egl-3(ok979)* worms responded like wild-type to the acute effects of ethanol they did not exhibit ethanol withdrawal (see Video S5, S6 and S7 for examples).

One possible explanation for the absence of ethanol withdrawal in *egl-3* mutant worms is that the behavioural response to chronic ethanol exposure might require a down-regulation of neuropeptide signalling. Such a mechanism has been suggested for NPY signalling in rodents conditioned on ethanol [51]. If this was occurring in *C. elegans* then it might be expected that *egl-3* itself would phenocopy aspects of ethanol withdrawal. Indeed in food races we observed that *egl-3* ethanol naive worms performed poorly (over a 2 h period, less than 5 out of 50 worms reached the food), similar to wild-type experiencing withdrawal. However, *egl-3* mutants did not show increased unaccompanied omega turns (no unaccompanied omega turns in 10 ethanol naive *egl-3* worms; Video S5) and therefore do not phenocopy ethanol withdrawal. Therefore a model that better fits the data is that alcohol triggers release of neuropeptide and this is required during, and (or) after, the conditioning period for ethanol withdrawal.

To confirm the role of *egl-3* in ethanol withdrawal we re-introduced wild-type *egl-3* into the *egl-3* mutant background by cosmid injection. This rescued the behaviour of unaccompanied omega turns following ethanol conditioning (Figure 12; p<0.05; Video S8). Therefore, a normally rare behaviour, unaccompanied omega turns is significantly and specifically increased in worms experiencing ethanol withdrawal, relieved by a low dose of ethanol, and the induction of this behaviour is dependent on peptidergic signalling.

## Discussion

In order to facilitate genetic understanding of the neuroactive properties of alcohol we have devised a new approach for studying ethanol withdrawal in *C. elegans*. An important aspect of this study is that we used the observation of withdrawal relief as a criterion to discriminate between adaptations directly caused by alcohol, and adaptations resulting indirectly from the pleiotropic effects of alcohol on behaviour. The rationale for this is based on the hypothesis that a low dose of alcohol relieves withdrawal by resetting the balance of signalling in neural circuits that have undergone ethanol-induced allostatic and, or, homeostatic adaptation [3], [4]. Thus only those behaviours which are a genuine expression of alcohol-induced neuroadaptation would be predicted to be subject to withdrawal relief.

The assay for withdrawal and withdrawal relief developed here was based on a food race assay. In this assay, *C. elegans* that were either acutely exposed to ethanol, or chronically exposed and then withdrawn were shown to have a reduced ability to navigate towards food. More detailed analysis of this impairment indicated that for acutely exposed worms this primarily resulted from a reduction in the speed and efficiency of movement while the pattern of locomotory behaviour in terms of the frequency of reversals was relatively unaffected. In marked contrast, worms in ethanol withdrawal appeared severely uncoordinated and exhibited reduced reversal frequency and an increased frequency of omega turns in the first 5 min in a food race task.

We next considered the different components of the withdrawal behaviour i.e. the effect on reversals and on omega turns, to determine whether or not either of these was likely to reflect direct ethanol-induced neuroadaptation. The decrease in reversal frequency was not relieved by a low dose of ethanol and may, at least in part, be explained by an adaptation to food deprivation brought about by ethanol inhibition of pharyngeal pumping [18], [28]. This is supported by the rather surprising observation that food deprivation also impaired performance in the food race. However, previous reports indicate that food deprivation inhibits reversal frequency [38] and this provides an explanation for poor navigational performance.

The second component of withdrawal behaviour we describe here was the expression of exaggerated body postures. This consisted primarily of a behaviour termed omega turns. In wild-type worms these high angled bends are tightly coupled to reversals in movements called ‘pirouettes’ during exploratory behaviour [36]. In ethanol withdrawal, omega turns occurred independently of reversals, leading to unaccompanied omega turns. Previous studies have indicated that unaccompanied omega turns are a rare behaviour in wild-type animals [37] and, furthermore, we are not aware of reports of mutants that exhibit this behaviour. The occurrence of unaccompanied omega turns is not simply due to an uncoupling of reversals from omega turns because the absolute frequency of omega turns and the overall posture measure, which indicates bendiness, were both increased. Furthermore, this behaviour was specific to ethanol withdrawal and was not seen during food deprivation. Most importantly, it was ameliorated by a low dose of ethanol. From this we deduce that it is an adaptive behaviour that is expressed when ethanol is withdrawn from worms that have been chronically exposed to ethanol for 6 h or longer. Worms which had developed withdrawal also showed tolerance to a high dose of ethanol, consistent with observations in other animals including humans that these phenomena are inter-related.

The occurrence of unaccompanied omega turns in withdrawal, and the relief of this by a low dose of ethanol, suggests that chronic ethanol exposure has altered the balance of signalling in the command circuits that regulate locomotory pattern during navigational behaviour. Some insight into the neural pathways that might be affected is provided by a study detailing the circuitry that drives reversals and omega turns during exploration [37]. These behaviours are differentially regulated by distinct motor circuits. The head and neck motorneurones, SMD and RIV, direct omega turns whilst the forward and backward command interneurones control reversals [37]. Intriguingly, laser ablation of the glutamatergic reverse command interneurone AVA resulted in worms that exhibited omega turns in the near complete absence of reversals i.e. unaccompanied omega turns [37] and thus superficially would appear to phenocopy this aspect of ethanol withdrawal. Further neurones of interest are the head motorneurones, SMB, SMD and RIV. Laser ablation of SMB increases the amplitude of dorsal-ventral head turns leading to very loopy movement whilst laser ablation of SMD and RIV has the opposite effect leading to a decrease in omega turns [37]. This suggests that these motorneurones play a key role in regulating the amplitude of head flexion required to make turns. Thus in ethanol withdrawal the output from SMB, SMD and RIV may be altered. These observations highlight the opportunity for an integrative analysis of adaptive responses to ethanol provided by defining withdrawal in a genetically tractable animal in which the circuits driving sub-behaviours are relatively simple and delineated.

As a first step to interrogating the genetic basis of withdrawal behaviour in *C. elegans* we investigated whether mutants previously reported to affect ethanol dependent behaviours altered responses in the food race paradigm. However, neither the NPY-like peptide receptor, NPR-1, previously reported to phenocopy the behaviour of *C. elegans* in ethanol withdrawal [17] nor the BK potassium channel SLO-1, previously reported as ethanol resistant [15], showed a markedly different profile of ethanol responses compared to wild-type.

There is a consensus that peptidergic pathways come in to play particularly when an animal is subject to environmental stressors [52] and neuropeptides are strong candidates as ethanol effectors in addiction [53] therefore we further investigated the role of neuropeptides using a more inclusive approach i.e. by employing *egl-3(ok979)*, a putative null for a neuropeptide precursor convertase enzyme [49] and severely depleted in neuropeptides [50]. Interestingly, this mutant did not exhibit withdrawal but was affected by acute exposure to ethanol. The phenotypes we observed for *egl-3* were otherwise relatively mild, similar to earlier studies [54], and serve to illustrate the neuromodulatory role of neuropeptides.

A further consideration is the dose-dependence of the effects of ethanol. In this study the lowest concentration that gave rise to withdrawal signs was around 136 mM, and for relief was 50 mM. These concentrations were applied externally and it has previously been reported that the internal concentration is ten fold lower than the external [15], [17], [18], [28]. However, observations on the fast kinetics of ethanol effects in intact worms [14], [18] and the similar sensitivity of exposed and intact tissue to ethanol [18] conflict with this and suggest that equilibration of ethanol across the worm cuticle is likely to be rapid. Therefore we predict that the internal concentration of ethanol approximates to the external concentration. Importantly, the rapid kinetics of the ethanol effects makes it impossible to accurately determine the internal concentration using a biochemical assay and so this issue remains to be resolved.

In terms of the withdrawal relief experiments, we observed an effect at 50 mM ethanol. On the basis of the argument outlined in the paragraph above, we predict that the tissue concentration in the worm in these experiments is very close to 50 mM. It could be argued that the relieving concentration of ethanol, 50 mM, is additive to ethanol remaining inside the worm following the ethanol conditioning procedure. However, evidence from our own studies and the literature indicate this is unlikely to be the case. Thus whilst there is some dispute concerning the internal concentration of ethanol in worms following immersion in a known external concentration [15], [18] an aspect that all the publications agree on is that if you take a worm that has been placed in a high concentration of ethanol (300 to 400 mM) and then rapidly collect, cool, wash and homogenise the worms then the concentration of ethanol measured in the worms is approximately 20 mM [15], [17], [18], [28]. Therefore, as we included an extended wash procedure, with no cooling, we can predict that the concentration remaining inside the worm after ethanol conditioning and washing is less than 20 mM. Thus, in the withdrawal relief experiments conducted at 50 mM ethanol it is unlikely that the tissue concentration is much in excess of this value. On this basis, the ability to induce a withdrawal phenomenon that was subject to ethanol relief with 50 mM ethanol indicates that *C. elegans* can exhibit responses to ethanol within a range that is sub-lethal in higher animals, including human.

**Figure 13:**
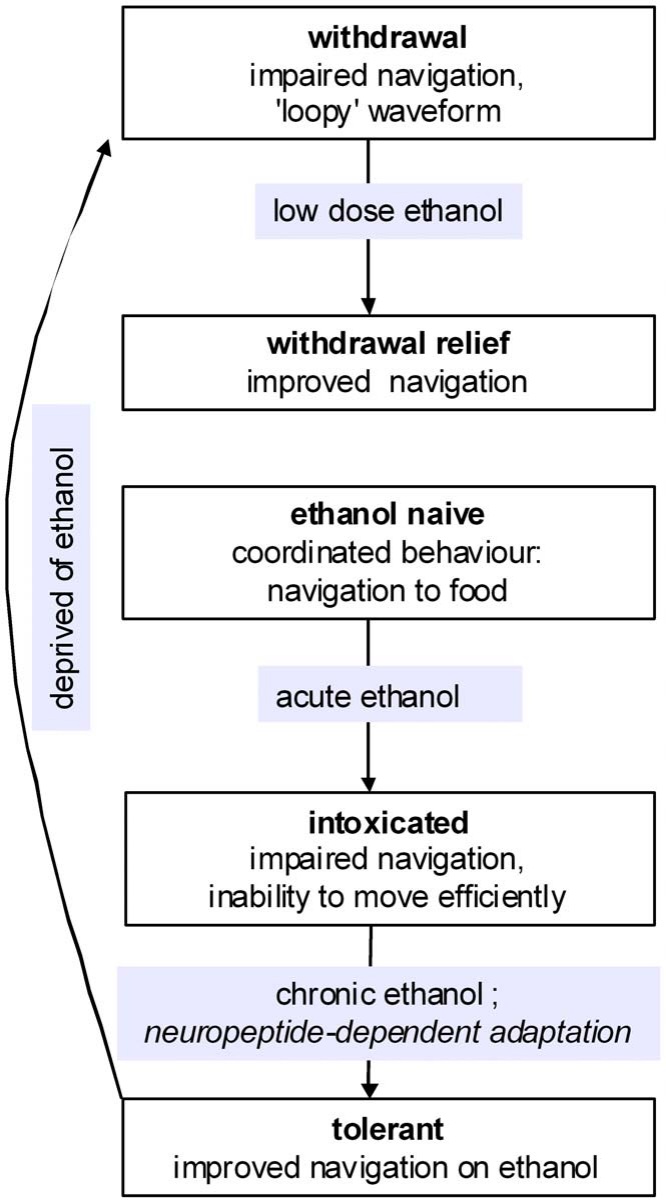
A model to describe the effects of acute and chronic ethanol on the perfomance of *C. elegans* in the food race task.

In conclusion, this study demonstrates the phenomenon of ethanol withdrawal and withdrawal relief in *C. elegans*. Whilst this simple animal system cannot model complex aspects of human alcohol addiction such as motivation, craving and cue-dependent relapse [55]–[57] it can provide a reductionist correlate of ethanol-induced neural plasticity which underpins negative reinforcement and therefore contributes to alcohol addiction. By defining, and investigating the genetic basis for, distinct but inter-related ethanol-induced behavioural states in *C. elegans* (Figure 13) we have shown that neuropeptide signalling has a pivotal role in ethanol withdrawal. Further studies are required to identify the precise neural substrate of this adaptive response in *C. elegans*. In particular, it will be very informative to identify which *C. elegans* neuropeptides and neuropeptide receptors are required for the *egl-3* dependent effect of alcohol withdrawal. Despite the fact that the *C. elegans* neuropeptides are a large and diverse family of approximately 250 molecules [45], [46] this is nonetheless a tractable problem given the molecular techniques that can be applied in this model system. As far as the receptors are concerned, there are 60 rhodopsin-like G protein-coupled receptors in *C. elegans* predicted to bind small molecule neurotransmitters or neuropeptides [48] and these are therefore candidate effectors for alcohol withdrawal. Within this group there are homologues of many of the mammalian neuropeptide receptor families [48] and further studies will be aimed at defining which of these are involved in *C. elegans* withdrawal. For the time-being we can note a key role for neuropeptides in this phenomenon. This provides a tantalizing parallel with mammalian studies in which neuropeptides have also been implicated in chronic responses to ethanol, including NPY [41], CRF [30], the opioid peptides [42], [43] and tachykinins [58]. This reinforces the importance of conserved pathways consistent with current investment in the development of drugs targeting peptidergic systems for the treatment of alcoholism [29], [59]. A recent focus of interest comes from data from mammalian studies which implicate hyperactivity in the extra-hypothalamic neuropeptide corticotrophin-releasing factor (CRF) signalling pathway in behavioural neuroadaptation to ethanol and stress-induced reinstatement of alcohol consumption [31], [32], [33], [34]. A seductive model is one in which chronic ethanol acts as a stressor to trigger neuropeptidergic signalling and neural plasticity in an evolutionarily conserved manner.

## Materials and Methods

### *C. elegans* maintenance and strains

Maintenance and manipulation of *C. elegans* were as previously described [60]. Strains used were: N2 (Bristol) strain for wild-type, AX201 *npr-1*(*ky13*), XA3441 *egl-3(ok979)*; XA3747 *slo-1(pd23)*, XA3748 *slo-1(pd24)*.

### Cosmid rescue

Adult hermaphrodites (XA3441) were injected with 10 ng µl^−1^ cosmid C26B6 with 50 ng µl^−1^ *Pmyo2::gfp* as a transformation marker. This drives expression of wild-type *egl-3* from the native *egl-3* promoter. Transformants were selected as F2 expressing gfp in the pharynx.

### Food race assays

Conditioning plates were made as follows: 6 cm NGM plates were seeded with 50 µl of *E. coli* OP50 at an optical density of 0.8 A (OD600) 3 days prior to use. One day prior to use, ethanol was added to half the plates, and they were sealed, to give the desired concentration. For high dose ethanol plates and conditioning plates, ethanol was added to give a predicted concentration of 200 to 300 mM and for low dose ethanol plates, ethanol was added to give a predicted concentration of 50 mM. Ethanol concentration measurements are given either as the predicted concentration or the measured value [made as previously described, 15]. Visual observation indicated that the growth of the bacterial lawn was not affected in these conditions. To make food race plates, 9 cm NGM plates were poured three days prior to use. They were seeded with *E.coli*, as before, 2 cm from the edge of the plate 2 days prior to use. Ethanol was added as described above. For the preconditioned food race, L4 worms were picked onto the conditioning plates (50 worms per plate) and left for the conditioning period (2 to 48 h). They were then washed twice for at least 2 min in M9 to remove residual ethanol [12] and then added to the food race plates 2 cm from the edge opposite to the food. The number of worms on the food was recorded every 10 min and the worms on the food were removed. After 2 h the number of worms remaining on the plate was recorded.

### Behavioural analysis of individual worms

All behavioural analysis was performed on age synchronized worms (L4 + 1 day or L4 + 2 days, as indicated) 5 or 40 min after placing them on food race plates. Experiments were conducted in parallel for all experimental conditions on the same day. There was no significant difference between 1 day old and 2 day old adults in food race performance (95±5%, n = 3, reached the food source by the 2 h time-point for L4 plus 1 day worms, and 93±4% for L4 plus 2 days.) Behaviours were defined as previously described [37]. For video capture, worms were picked onto conditioning or control plates and left for 6 h. They were then individually washed for 2 min in M9 to remove residual ethanol and placed onto a non-food plate for 1 min to remove excess liquid, before being picked onto a food race plate, 2 cm from the edge opposite the food and left for 5 min. After 5 min a 30 second video was recorded of the behaviour of the worm. Video recordings were taken using a dissecting microscope attached to a Hamamatsu C4742-95 camera and using SimplePCI video recording software. All videos were taken at the same magnification and are shown at 2x actual speed.

### Automated off-line analysis of videos

The automated video analysis was carried out using a software package written in Matlab. Each video is a grey-scale .avi video file containing up to 150 frames showing a single worm moving on agar. Each frame is a rectangle of 1024×1280 pixels, each pixel of which has been assigned a value for intensity, which describes its colour along a grey scale between black (0) and white (255). To extract the background image the assumption was that the worm was the only thing moving in the video. Thus to extract the background the mean image was taken of the 150 frames. Every pixel has a value for its intensity in each of the 150 frames. The average of these values was assigned to that pixel to create an average image. The worm will be much darker (lower intensity) than its surroundings. But, as it moves around, it will be averaged out of the background image. The operator is then shown the background image and asked if this is correct. If the worm has remained stationary it will still be visible. If this is the case the operator can draw a rectangle around the area where the worm is still visible. The program will then constructs a histogram of the intensity values within this rectangle. There will be two peaks on the histogram, one corresponding to the worm and one to the background. The program will calculate the median intensity value and replace all the intensity values below the median with the median value. This will remove the worm from the background image. The operator is then shown the new background image and asked if this is correct. This process can repeat until the operator is satisfied. In order to create a binary image of the worm the program works on a frame by frame basis. The background image is subtracted from each frame. Pixels that contain the background should thus have an intensity value close to zero. All pixels with intensity values that are within a certain range of zero are assigned the value zero (black). All pixels with intensity values beyond this threshold are assigned the value one (white). A binary image of the worm has thus been produced. A parameterized worm model was constructed on a frame by frame basis. The parameterised worm was described using a Gaussian mixture model (GMM). The frame of the video being analysed can be described as a 3D graph with the co-ordinates of each pixel in the 2D frame along the x and y axes and the intensity of that pixel on the z axis. This produces a distribution which cannot be easily described statistically. This is modelled using ten Gaussian distributions, which are well characterised statistically. Each Gaussian can be described by its mean (x and y coordinates), amplitude (intensity) and variance. In order to model the worm using these ten Gaussians, an expectation maximisation (EM) paradigm is used. This measures the error between the Gaussian mixture model and the actual intensity distribution of the image, alters the parameters of the Gaussians and measures the error again. This repeats until the error converges. This paradigm minimises the error between the model and the real image. To minimise the number of iterations required constraints are placed on the amplitude and variance of the distributions and the initial mean coordinates are taken from the final mean coordinates of the previous frame. For the first frame in the video the operator is shown a binary image of the worm from the first frame and asked to mark ten points along its length with the mouse, starting with the head to give the initial coordinates. The ten Gaussians are numbered 1–10 in accordance with the order in which the operator marked them in the first frame, with 1 being the head of the worm and 10 being the tail. A parameterised worm is drawn by taking the ten mean x, y coordinates of the Gaussians (node centres) and joining them with a best fit curve. The head is marked in red. The x, y coordinates of the ten node centres in every frame of the video are then saved. The degree of bendiness of the worm, called the posture measure, is determined by a linear regression line drawn between the ten node centres. This is the straight line which minimises the sum of the squares of the perpendicular distances of each node centre to the line. This is not affected by the ordering of the node centres. The perpendicular distance of each node centre to the regression line is then measured and the ten values averaged. This gives a value for the bendiness of the worm for each frame. The value for every frame in the video can be averaged to give an overall value called the posture measure. The higher the value, the more loopy or bendy the behaviour of the worm. The centre of mass of the worm was calculated as follows. Each of the ten node centres has an x and a y coordinate. The average of all the x coordinates is the x coordinate of the centre of mass and likewise for the y coordinates. This gives an x, y coordinate for the centre of mass of the worm in any given frame. By joining the centre of mass position for every frame in the video a track of the movement of the worm can be drawn. The centres of mass can be used to calculate the distance travelled by the worm between each frame and thus the total distance travelled during the video. This distance divided by the duration of the video (in seconds) is the average speed of the worm. An alternative centre (centre of worm) is calculated by measuring half the distance of the length of the best fit curve joining the ten node centres, along the best fit curve joining the ten node centres. If the centre of the worm in every frame is joined up to make a track, this can also be used to calculate distance travelled. This produces a larger value as this track follows the sinusoidal movement of the worm. These two values for distance travelled, distance covered by centre of mass (d1) and distance covered by centre of worm (d2), can be used to define the efficiency of movement (by dividing d1 by d2).

### Statistical analysis and data presentation

Data are presented as mean ± s.e.mean. One way or two way ANOVA with Bonferroni's Multiple Comparison Test were applied to data sets as appropriate (GraphPad Prism, version 4, La Jolla, CA 92037). Statistical significance was assumed at p<0.05. * indicates p<0.05, ** p<0.01 and *** p<0.001.

To aid interpretation, the same colour code is used to represent the ethanol treatment groups (for all the relevant figures except figure 11): ‘naive’, (not pre-exposed to ethanol and tested in the absence of ethanol; black symbols); ‘naive + low eth’, (not pre-exposed to ethanol and tested in the presence of 50 mM ethanol; grey symbols); ‘naive + high eth’, (not pre-exposed to ethanol and tested in the presence of 250 mM ethanol; magenta symbols); ‘withdrawn’, (conditioned in 250 mM ethanol and tested in the absence of ethanol; red symbols); ‘withdrawn + low eth’, (conditioned in 250 mM ethanol and tested in the presence of 50 mM ethanol; green symbols); ‘withdrawn + high eth’, (conditioned in 250 mM ethanol and tested in the presence of 250 mM ethanol; blue symbols).

## Supporting Information

Figure S1 Automated analysis of *C. elegans* behaviour on the food race plate. Illustration of the automated off-line analysis of videos. A. Process of extracting the position of the parameterised worm from a video frame. Clockwise from top left; the mean background image, one frame with the background deleted, the binary image of the same frame and lastly, also from the same frame, the best fit curve between the ten node centres with node 1 (the head) marked in red. B. Regression line through ten node centres in one frame of a video from which the posture measure is calculated. C. Track showing the position of the centre of mass of the worm in every frame of a single video from which speed is calculated. Colour scale shows posture measure. D. Track showing the positions of the centre of the worm (blue) and the centre of mass (pink) in every frame of the video. These are the measures from which efficiency is calculated.
(0.13 MB TIF)

Video S1 Wild-type ethanol naive. Note that the reversal is coupled to an omega turn. Video recordings were taken using a dissecting microscope attached to a Hamamatsu C4742-95 camera and using SimplePCI video recording software. All videos were taken at the same magnification after 5 min on the food race plate and are played back at 2 x normal speed.
(0.65 MB AVI)

Video S2 Wild-type in the presence of 250 mM ethanol. Video recordings were taken using a dissecting microscope attached to a Hamamatsu C4742-95 camera and using SimplePCI video recording software. All videos were taken at the same magnification after 5 min on the food race plate and are played back at 2 x normal speed.
(0.40 MB AVI)

Video S3 Wild-type that has been chronically exposed (6 h) to 250 mM ethanol and then tested in the food race in the absence of ethanol, ‘withdrawn’. Note the unaccompanied omega turn. Video recordings were taken using a dissecting microscope attached to a Hamamatsu C4742-95 camera and using SimplePCI video recording software. All videos were taken at the same magnification after 5 min on the food race plate and are played back at 2 x normal speed.
(0.60 MB AVI)

Video S4 A second example of wild-type that has been chronically exposed (6 h) to 250 mM ethanol and then tested in the food race in the absence of ethanol, ‘withdrawn’. Note that in this worm the reversal is followed by the worm curling up into a tight ball. Video recordings were taken using a dissecting microscope attached to a Hamamatsu C4742-95 camera and using SimplePCI video recording software. All videos were taken at the same magnification after 5 min on the food race plate and are played back at 2 x normal speed.
(0.77 MB AVI)

Video S5 An ethanol naive egl-3(ok979) mutant. Video recordings were taken using a dissecting microscope attached to a Hamamatsu C4742-95 camera and using SimplePCI video recording software. All videos were taken at the same magnification after 5 min on the food race plate and are played back at 2 x normal speed.
(0.99 MB AVI)

Video S6 An egl-3(ok979) mutant tested in the presence of 250 mM ethanol. Video recordings were taken using a dissecting microscope attached to a Hamamatsu C4742-95 camera and using SimplePCI video recording software. All videos were taken at the same magnification after 5 min on the food race plate and are played back at 2 x normal speed.
(0.99 MB AVI)

Video S7 An egl-3(ok979) mutant tested that has been chronically exposed (6 h) to 250 mM ethanol and then tested in the food race in the absence of ethanol, ‘withdrawn’. Note the absence of unaccompanied omega turns. Video recordings were taken using a dissecting microscope attached to a Hamamatsu C4742-95 camera and using SimplePCI video recording software. All videos were taken at the same magnification after 5 min on the food race plate and are played back at 2 x normal speed.
(1.00 MB AVI)

Video S8 An egl-3(ok979) mutant that is expressing a wild-type copy of egl-3 from the native promoter. This worm had been chronically exposed (6 h) to 250 mM ethanol and then tested in the food race in the absence of ethanol, similarly to the worm shown in Video S7. Note the marked occurrence of high-angled body bends and unaccompanied omega turns. Video recordings were taken using a dissecting microscope attached to a Hamamatsu C4742-95 camera and using SimplePCI video recording software. All videos were taken at the same magnification after 5 min on the food race plate and are played back at 2 x normal speed.
(0.63 MB AVI)
