# Supplementary figures and images for: A Differential Role for Neuropeptides in Acute and Chronic Adaptive Responses to Alcohol: Behavioural and Genetic Analysis in Caenorhabditis elegans
Source: PLoS One. 2010 May 3;5(5):e10422. doi: 10.1371/journal.pone.0010422 (PMC2862703; doi:10.1371/journal.pone.0010422)

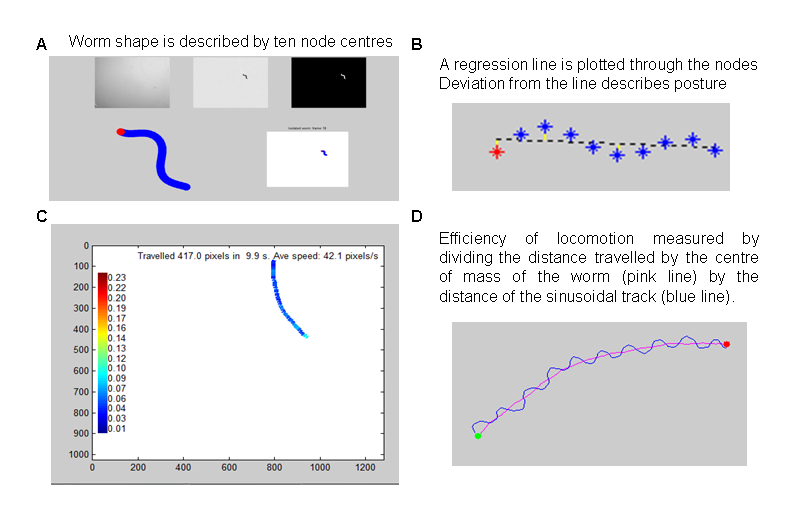

Supplement: Figure S1 — Automated analysis of C. elegans behaviour on the food race plate. Illustration of the automated off-line analysis of videos. A. Process of extracting the position of the parameterised worm from a video frame. Clockwise from top left; the mean background image, one frame with the background deleted, the binary image of the same frame and lastly, also from the same frame, the best fit curve between the ten node centres with node 1 (the head) marked in red. B. Regression line through ten node centres in one frame of a video from which the posture measure is calculated. C. Track showing the position of the centre of mass of the worm in every frame of a single video from which speed is calculated. Colour scale shows posture measure. D. Track showing the positions of the centre of the worm (blue) and the centre of mass (pink) in every frame of the video. These are the measures from which efficiency is calculated. (0.13 MB TIF) [file pone.0010422.s001.tif]
